# Determinants of Adherence to World Cancer Research Fund/American Institute for Cancer Research Recommendations in Women with Breast Cancer

**DOI:** 10.3390/cancers17040708

**Published:** 2025-02-19

**Authors:** Vanessa Pachón Olmos, Marina Pollán, Nerea Fernández de Larrea-Baz, Julia Fernández-Morata, Emma Ruiz-Moreno, Javier García-Pérez, Adela Castelló, María Ángeles Sierra, Pilar Lucas, Isabel Alonso-Ledesma, Agostina Stradella, Blanca Cantos, Teresa Ramón y Cajal, Marta Santisteban, Miguel Ángel Seguí, Ana Santaballa Bertrán, Mónica Granja, Julia Camps-Herrero, Sabela Recalde, Miriam Mendez, Nuria Calvo Verges, Beatriz Pérez-Gómez, Roberto Pastor-Barriuso, Virginia Lope

**Affiliations:** 1 Preventive Medicine Department, La Paz-Carlos III-Cantoblanco University Hospital, Po de la Castellana 261, 28046 Madrid, Spain; 2 Cancer and Environmental Epidemiology Unit, Department of Epidemiology of Chronic Diseases, National Center for Epidemiology, Instituto de Salud Carlos III (ISCIII), Avda. Monforte de Lemos 5, 28029 Madrid, Spainacastello@isciii.es (A.C.); isabel.alonso@isciii.es (I.A.-L.);; 3 Consortium for Biomedical Research in Epidemiology and Public Health (CIBERESP), Avda. Monforte de Lemos 3-5, 28029 Madrid, Spain; 4 Programa de Doctorado en Ciencias Biomédicas y Salud Pública IMIENS-UNED-ISCIII, Escuela Internacional de Doctorado de la Universidad Nacional de Educación a Distancia (EIDUNED), Avda. Monforte de Lemos 5, 28029 Madrid, Spain; 5 Multidisciplinary Breast Cancer Unit, Department of Medical Oncology, Institut Català d’Oncologia, Idibell, Avinguda de la Granvia de l’Hospitalet, 199, 08908 L’Hospitalet de Llobregat, Spain; 6 Department of Medical Oncology, Hospital Universitario Puerta de Hierro, C. Joaquín Rodrigo, 1, 28222 Majadahonda, Spain; 7 Consulta de Cáncer Familiar, Department of Medical Oncology, Hospital de la Santa Creu i Sant Pau, Carrer de Sant Quintí, 89, Horta-Guinardó, 08025 Barcelona, Spain; 8 Breast Cancer Unit, Department of Medical Oncology, Clínica Universidad de Navarra, IdiSNA, Av. de Pío XII, 36, 31008 Pamplona, Spain; 9 Department of Medical Oncology, Consorcio Hospital Universitario Parc Tauli, Parc Taulí, 1, 08208 Sabadell, Spain; 10 Medical Oncology Department, La Fe Health Research Institute (IISLaFe), La Fe University Hospital, Avinguda de Fernando Abril Martorell, 106, Quatre Carreres, 46026 Valencia, Spain; 11 Department of Medical Oncology, Hospital Universitario Clínico San Carlos, Calle Prof Martín Lagos, S/N, Moncloa-Aravaca, 28040 Madrid, Spain; 12 Department of Radiology, Hospital Universitario de La Ribera, Ctra. Corbera km 1, 46600 Alzira, Spain; 13 Multidisciplinary Breast Cancer Unit, Department of Medical Oncology, Institut Català d’Oncologia-Hospitalet, Avinguda de la Granvia de l’Hospitalet, 199, 08908 L’Hospitalet de Llobregat, Spain

**Keywords:** lifestyle recommendations, WCRF/AICR guidelines, cancer prevention recommendations, compliance, health behaviors, healthy lifestyle, breast cancer patients

## Abstract

Background/Objectives: The 2018 World Cancer Research Fund/American Institute for Cancer Research (WCRF/AICR) cancer prevention recommendations benefit primary prevention and survivor outcomes. This study evaluated the adherence to these recommendations during the year prior to breast cancer diagnosis and identified related clinical and sociodemographic factors. Methods: A total of 915 patients with breast cancer were recruited from eight hospitals in four regions of Spain. The participants completed an epidemiologic questionnaire and a food frequency questionnaire. The compliance with the WCRF/AICR recommendations was assessed using a standardized score based on seven recommendations. Standardized prevalences and standardized prevalence ratios (SPRs) for moderate and high adherence were calculated based on participant characteristics using binary and multinomial logistic regression models. Results: The mean adherence was 3.5 points out of 7. The recommendations with the best and worst adherence were avoiding sugar-sweetened drinks (54.4% adherence) and maintaining a fiber-rich diet (4.4% consumed ≥30 g/day). The overall adherence was better in women aged ≥60 years (SPR = 1.55; 95% CI = 1.09–2.22), and worse in those with a caloric intake ≥2000 kcal/day (SPR = 0.48; 95% CI = 0.37–0.62) or ≥2 comorbidities (SPR = 0.66; 95% CI = 0.49–0.89). The adherence to maintaining a healthy weight was worse in those with ≥2 comorbidities and stage III-IV tumors. The physical activity adherence was worse in working women and those with ≥2 comorbidities. The alcohol restriction adherence was worse in smokers. Younger women, smokers and those with a low calorie intake were less adherent to the fruit/vegetable recommendation. The consumption of fiber and limited consumption of red/processed meat adherence was poor in all the subgroups. The adherence to a limited consumption of fast food and sugary drinks was worse in younger women and high-calorie-diet consumers. Conclusions: The differences in the adherence to recommendations according to patient characteristics justify the design of personalized interventions for breast cancer patients.

## Simple Summary

International guidelines for cancer prevention, related to diet, physical activity and weight control, have demonstrated benefits both in primary prevention and in improving health outcomes after cancer diagnosis. Few studies have assessed the adherence to individual recommendations according to sociodemographic characteristics and tumor-related factors. This study analyzes the degree of adherence to the 2018 WCRF/AICR cancer prevention recommendations during the year prior to breast cancer diagnosis and identifies potentially influential factors in 915 Spanish patients. The overall adherence was moderate, being lower for maintaining a fiber-rich diet and higher for avoiding sugar-sweetened drinks. Younger women, those with a high calorie intake and those with more comorbidities displayed a poorer overall adherence, although the adherence to individual recommendations varied according to specific sociodemographic and clinical factors. Understanding the baseline situation regarding healthy habits in this population is crucial for developing programs aimed at improving these habits after diagnosis.

## 1. Introduction

Breast cancer was the most frequently diagnosed cancer in women in 2022, worldwide [1] and in Europe (EU-27), with an estimated age-standardized rate (European 2013 population) of 147.6 cases per 100,000 women [2]. In Spain, breast cancer also ranked first among diagnosed tumors in women in 2022, with 130.8 cases per 100,000 women (30.3% of all malignant tumors except non-melanoma skin cancer in women) [2]. The same year, Spain showed a standardized mortality rate of 23.3 deaths per 100,000 women (14.7% of all cancer deaths in Spain) [2]. In terms of survival, Spain has been improving in recent years, with the age-standardized 5-year net survival being 85.2% in women diagnosed in the period 2010–2014 [3]. The increase in survival has contributed to positioning this tumor as the most prevalent in Spain, reaching 516,827 cases as of December 2020 [4].

The second World Cancer Research Fund/American Institute for Cancer Research (WCRF/AICR) expert report published, in 2007, the 10 Cancer Prevention Recommendations, which focus on eating a healthy diet, maintaining a healthy body weight, and engaging in regular physical activity. These recommendations were updated in 2018 [5]. Increasing evidence demonstrates the benefits of maintaining healthy lifestyles not only in the primary prevention of breast cancer [6,7] but also in health outcomes after cancer diagnosis, such as the all-cause mortality [8], quality of life [9], tolerance to treatments [10] and others [11]. Therefore, the WCRF/AICR expert panel advises individuals to follow these recommendations as far as possible after cancer diagnosis [5].

While the interest in the benefits of adopting healthy lifestyles is growing, evidence indicates that the compliance with recommendations is low among cancer survivors, and that it may decrease with time since diagnosis [12,13]. Studies that delve into analyzing the sociodemographic characteristics that may influence this compliance are scarce [14,15], and its correlation with tumor stage or other anatomopathological and treatment-related factors is not often addressed [5]. Therefore, the aim of this study is to quantify the adherence to the 2018 WCRF/AICR recommendations before diagnosis in patients with breast cancer, and to identify sociodemographic and clinical factors linked to such adherence. Knowing the level of adherence before diagnosis and the associated factors would enable more precise targeting of recommendations and more personalized support for women with breast cancer.

## 2. Materials and Methods

### 2.1. Study Population

This study is part of a multicenter case–case project, the Breast Cancer & Density Association Study (BCDAS). A total of 1021 breast cancer patients were ambispectively recruited from the oncology departments of eight hospitals located in four Spanish regions (Catalonia, Madrid, Valencian Region and Navarre). Women diagnosed between January 2014 and March 2019 were interviewed between October 2016 and December 2019. Inclusion criteria were being over 18 years old, having a histological diagnosis of breast carcinoma (invasive or in situ) and being able to respond to an epidemiological questionnaire. Exclusion criteria included surgical interventions on the healthy breast before diagnosis with removal of breast tissue, breast reconstruction, breast augmentation and the presence of a synchronous bilateral tumor.

Recruitment was carried out by medical oncologists after obtaining written informed consent from the patients.

The protocol adhered to the principles of the Declaration of Helsinki and was approved by the Research Ethics and Animal Welfare Committee of the Instituto de Salud Carlos III (CEI PI 32_2015-v2, June 2015). Patient information was anonymized in the database before statistical analysis.

### 2.2. Study Variables

Trained interviewers administered an epidemiological survey by telephone. The survey included information on sociodemographic variables, anthropometric data, personal and family medical history, hormonal and reproductive information, alcohol consumption, tobacco use and physical activity. After recruitment, hospital researchers completed a questionnaire on clinical and pathological data.

Dietary intake during the year prior to diagnosis was estimated using a food frequency questionnaire, included in the epidemiological questionnaire, designed for the Spanish population and used in previous studies [16]. Responses for each food item were converted into mean daily or weekly intake (in grams) and total energy intake (in kilocalories/day), using the ALEVINT 1.0 software [17]. Physical activity was assessed in a specific section of the questionnaire that collected information on the practice of different types of sports and activities in the year prior to diagnosis, measured in hours per week, as well as the months during which each activity had been practiced. The total minutes/week of physical activity for each participant was calculated by adding up the annual average minutes/week dedicated to each activity.

The possible determinants of adherence to the 2018 WCRF/AICR recommendations evaluated were as follows: sociodemographic factors at diagnosis (age, recruiting region, educational level and working status), lifestyle factors at diagnosis (smoking status, parity and energy intake), clinical characteristics at diagnosis (menopausal status, number of comorbidities, emotional stress level, personal history of cancer and carrying a breast cancer-predisposing mutation (*BRCA1*, *BRCA2*, *PTEN*, *TP53*, *PALB2* and others)), family history of breast cancer and cancer diagnosis-related factors (years since diagnosis, molecular subtype and stage at diagnosis according to the 7th edition of the American Joint Committee on Cancer (AJCC) TNM staging system [18]). For tumor stage codification, pathological stage was prioritized over clinical stage, except in cases of neoadjuvant treatment or no surgical treatment.

### 2.3. Construction of the WCRF/AICR Compliance Score

The score of adherence to the 2018 WCRF/AICR prevention recommendations in the year prior to diagnosis was created based on guidelines previously published by Shams-White et al., that did not include the general recommendation on supplement intake and the additional recommendation on cancer survivors due to operational redundancy [19,20]. We could not include the additional recommendation on breastfeeding, because this information was not available for all the participants. Therefore, the score was built from the following seven components: (1) be a healthy weight; (2) be physically active; (3) eat a diet rich in whole grains, vegetables, fruit and beans; (4) limit the consumption of “fast foods” and other processed foods high in fats, starches, or sugars; (5) limit the consumption of red and processed meat; (6) limit the consumption of sugar-sweetened drinks; and (7) limit alcohol consumption. Table 1 shows the operationalized variables and the corresponding scores. The method of scoring assigned 1 point when the recommendation was fully met, 0.5 points when the recommendation was partially met and 0 points when it was not met [19]. For the recommendation of eating a diet rich in whole grains, vegetables, fruit and beans, which has two sub-recommendations, the punctuation was halved to maintain a total maximum score of one point. On the other hand, although be a healthy weight also has two sub-recommendations, as information on waist circumference was not available, the score for Body Mass Index (BMI) was doubled, as recommended by Shams-White et al. [19]. Ultra-processed food (UPF) intake was estimated based on an adaptation of the NOVA classification system [21], grouping foods based on the nature, scope and purposes of the industrial processes they undergo. As recommended by the authors, this variable was divided into terciles instead of using absolute reference values. Also following Shams-White et al.’s proposal, cut-offs for the physical activity recommendation, measured in minutes/week, were obtained from Spanish national guidelines [22]. The final score was calculated by summing individual scores, and, therefore, it ranged from 0 (no recommendations met) to 7 (all recommendations fully met). Lastly, three compliance categories were created based on the tertiles of the distribution of the total score in the study population: low adherence (<2.5 points), moderate adherence (2.5–3.5), and high adherence (>3.5).

### 2.4. Statistical Analyses

Continuous variables were summarized using means and standard deviations, while absolute figures and percentages were used for categorical variables. To assess significant differences by categories of compliance, Pearson’s chi-square test was applied for categorical variables, while for continuous variables, the Wald test was used through simple linear regression models.

To control for confounding, the prevalences of low, moderate and high compliance with the full cancer prevention recommendations, during the year prior to diagnosis, by categories of each sociodemographic and clinical characteristic were standardized to the distribution of selected confounding factors in the overall sample of breast cancer patients, including age at diagnosis (<50, 50–60, >60 years), recruitment region (Catalonia, Madrid, Valencian Region, Navarre), educational level (primary education or less, high school/vocational training, university graduate), working status at diagnosis (no, yes), smoking status at diagnosis (no, yes), energy intake in the year prior to diagnosis (<1500, 1500–2000, >2000 kcal/day), parity (parous or nulliparous), family history of breast cancer (none, second degree only, first degree), number of comorbidities (0, 1, ≥2), menopausal status at diagnosis (pre/perimenopausal, postmenopausal), and years since diagnosis (<1, 1–2, 3–5 years). This standardization was accomplished by first fitting a multinomial logistic model relating the three compliance levels with each target characteristic and adjusting for the above confounding factors, and then calculating the average predicted probabilities of low, moderate and high compliance, as if every breast cancer patient were in each category of the target characteristic with their confounding factors unchanged [23,24]. We estimated the standardized prevalence ratios (SPRs) for moderate and high compliance, comparing each category of the target characteristic with the reference one, together with their 95% confidence intervals (95% CIs).

We used similar model-based standardization methods to estimate the standardized prevalences of compliance with each specific recommendation across categories of sociodemographic and clinical characteristics, but fitting a binary logistic model for compliance (yes, no) with the specific recommendation and further adjusting for the overall compliance score obtained from the remaining recommendations. The resulting SP and 95% CIs were represented in forest plots.

All analyses were performed using the statistical software STATA/MP 18.0 (StataCorp LLC, College Station, TX, USA) and R 4.3.1.

## 3. Results

From the initial sample of 1021 women with breast cancer, 60 participants were considered ineligible, 19 declined to participate, 17 reported implausible caloric intake (<750 or >4500 kcal/day), and 10 were excluded due to lack of information on one or more components of the score. Thus, the final study sample consisted of 915 women with breast cancer, with a mean age of 56.8 years (range 23–97 years) at the time of the interview. The interviews were conducted on average 1.1 years after diagnosis (range 0–5 years).

Table 1 shows the percentage of adherence to each of the WCRF/AICR recommendations. The one with the highest compliance was the limited consumption of sugar-sweetened drinks (54.4% of compliant women). On the contrary, the recommendation with the worst adherence was eating a diet rich in fiber (2.6% consumed at least 30 g/day of total fiber).

The sociodemographic and clinical characteristics at diagnosis, overall and by tertiles of the WCRF/AICR score, are described in Table 2. The mean age of the participants was 55.3 years (range 22–94). University education was completed by 32.1% of the patients. In the year prior to diagnosis, 60.1% of the women were employed, and 76.8% did not smoke. Additionally, 83% of the women had at least one child and 62.6% were menopausal at the time of diagnosis. The distribution of the tumor subtypes was as expected (68.6% luminal, 19.1% Her2+ and 12.3% triple-negative).

The mean adherence score to the recommendations was 3.5 out of 7 points (range 0.75–6.50), and the prevalence of full adherence to three or more recommendations/sub-recommendations was 45.5%. Women with lower compliance were younger (<50 years) and had a higher caloric intake (≥2000 kcal/day) (Table 2).

Table 3 presents the SPRs of moderate and high compliance with the 2018 WCRF/AICR recommendations. Compared to the women with an intake of less than 1500 kcal/day, the SP of high compliance was 29% lower among those with an intake between 1500 and 1999 kcal/day (SPR = 0.71; 95% CI = 0.59–0.86), and 52% lower in patients with an intake of at least 2000 kcal/day (SPR = 0.48; 95% CI = 0.37–0.62). Women reporting two or more comorbidities at diagnosis had a 34% lower compliance with the recommendations than those who did not have any (SPR = 0.66; 95% CI = 0.49–0.89). In contrast, the SP of high compliance was 55% higher in the women over 60 years old compared to those under 50 years (SPR = 1.55; 95% CI = 1.09–2.22).

**Figure 1:**
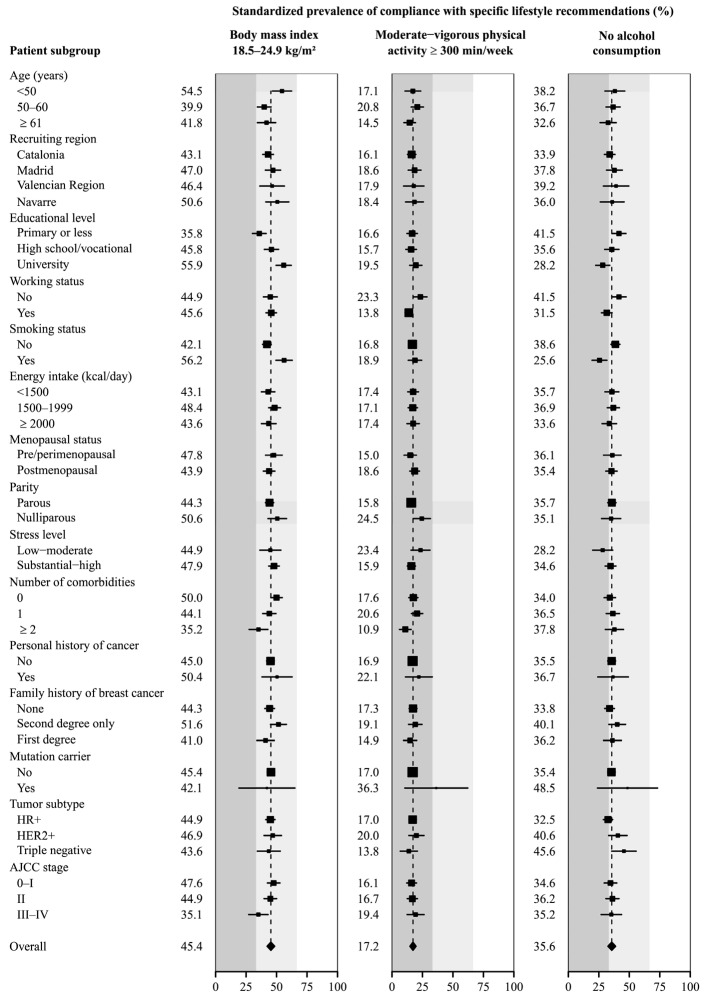
Standardized prevalence of compliance with specific WCRF/AICR lifestyle recommendations by participant characteristics at diagnosis. Prevalences (squares with area inversely proportional to their variances) and 95% confidence intervals (horizontal lines) were standardized to the overall distribution of age, recruiting region, educational level, working status, smoking status, caloric intake, menopausal status, parity, number of comorbidities, family history of breast cancer, years since diagnosis and the overall adherence to the other recommendations in the entire sample of breast cancer patients. Column colors correspond to tertiles of total adherence score.

**Figure 2:**
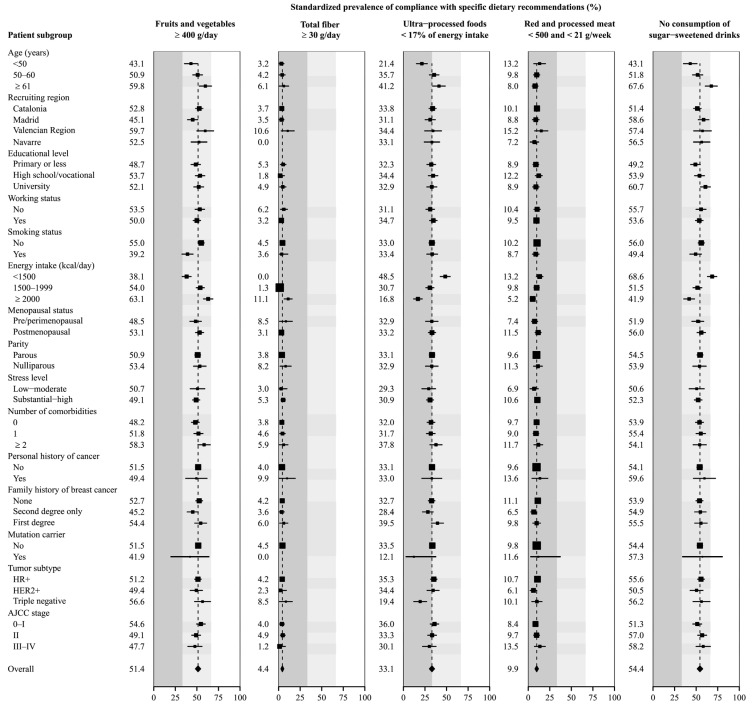
Standardized prevalence of compliance with specific WCRF/AICR dietary recommendations by participant characteristics at diagnosis. Prevalences (squares with area inversely proportional to their variances) and 95% confidence intervals (horizontal lines) were standardized to the overall distribution of age, recruiting region, educational level, working status, smoking status, caloric intake, menopausal status, parity, number of comorbidities, family history of breast cancer, years since diagnosis and the overall adherence to the other recommendations in the entire sample of breast cancer patients. Column colors correspond to tertiles of total adherence score.

Figure 1 and Figure 2 show the SP and 95% CI of high compliance with each of the 2018 WCRF/AICR recommendations. The recommendations with the highest SP of high compliance were limited consumption of sugar-sweetened drinks (54.4% did not consume them, 95% CI = 51.4–57.5) and fruit and vegetable intake (51.4% consumed at least 400 g/day, 95% CI = 48.4–54.3). In contrast, fiber consumption was the recommendation with the lowest adherence (4.4% consumed at least 30 g/day, 95% CI = 2.7–6.0), followed by the limited consumption of red and processed meat, with only 9.9% of women (95% CI = 7.9–11.8) eating less than 500 g of red meat and less that 21 g of processed meat per week.

Figure 1 displays the results corresponding to the recommendations for BMI, physical activity, and alcohol consumption. For maintaining a healthy BMI, the overall SP of high adherence was 45.4% (95% CI = 42.3–48.4). Women under 50 years old, university-educated women and smokers showed an SP over that value, while the SP was lower among the less educated patients, women with two or more comorbidities and those with stage III-IV tumors. Concerning physical activity, the global SP of high compliance was 17.2% (95% CI = 14.8–19.7), and was higher among women that did not work and had no children and lower among those employed at the time of diagnosis or with two or more comorbidities. Regarding the recommendation to reduce alcohol consumption, 35.6% of the participants were non-drinkers (95% CI = 32.5–38.7). Less educated women, those who were not working, or those with triple-negative breast cancer presented a higher prevalence of compliance, while university-educated women and smokers showed lower compliance.

In terms of the compliance with dietary recommendations (Figure 2), the SP of high adherence for fruit and vegetable consumption was 51.4% (95% CI = 48.4–54.3). This prevalence was higher among women older than 61 years, non-smokers, those consuming 2000 Kcal/day or more and those with two or more comorbidities, while younger women, patients from the Madrid region, smokers and those with a lower caloric intake showed lower adherence. The adherence to fiber intake recommendation showed an SP of 4.4% (95% CI = 2.8–6.0), and was higher among patients who consumed at least 2000 Kcal/day and lower among those with second-degree education or with stage III-IV tumors at diagnosis. Regarding the fast food consumption, the overall prevalence of high adherence was 33.1% (95% CI = 30.2–36.0), showing lower prevalence in women younger than 50 years, those with a higher caloric intake, mutation carriers and women with triple-negative tumors. The compliance with the recommendation to limit the intake of red and processed meat was 9.9% (95% CI = 7.9–11.8), being lower among women consuming 2000 Kcal/day or more. Lastly, the SP of not consuming sugar-sweetened drinks was 54.5% (95% CI = 51.4–57.5), and was lower among younger women and in those with a higher caloric intake.

## 4. Discussion

The women in our study demonstrated moderate compliance with the 2018 WCRF/AICR recommendations (mean adherence score of 3.5 points out of 7). The recommendations with the highest adherence were those related to limiting the consumption of sugar-sweetened drinks, eating fruits and vegetables, maintaining a healthy weight, and limiting alcohol and fast food consumption, with between one-third and one-half of women being highly compliant with them. On the other hand, lower adherence was observed with the recommendations of following a diet rich in fiber and limiting red and processed meat intake, with less than 10% displaying high compliance. The overall compliance was lower among the younger women, those with a higher daily caloric intake and those with more than one comorbidity.

There is evidence regarding the association between adherence to the 2018 WCRF/AICR recommendations and breast cancer risk [6,7,25]. However, few studies have analyzed the compliance with each recommendation by sociodemographic and clinical characteristics [14,15]. This study examines the adherence to these recommendations at the time of cancer diagnosis. Knowing the baseline situation regarding healthy habits in this population is crucial to implement programs aimed at improving these habits after diagnosis, since they have been associated with health outcomes among survivors [8,9,26,27,28]. Previous studies have shown that the diagnosis itself motivates patients to make behavioral changes toward healthier lifestyles, but this motivation decreases over time [29], so the early stages after diagnosis seem to be a crucial time to implement these prevention and health promotion activities [12,29]. Furthermore, the increase in breast cancer survival, with a rise in the prevalence of survivors and greater social sensitivity towards healthy lifestyles [30], are additional compelling reasons to work on health promotion in this group [31]. Sociodemographic factors may influence lifestyle habits, as demonstrated in the study by Malcosom et al. [14]. In our study, several sociodemographic and clinical factors were also found to affect compliance with the WCRF/AICR recommendations, in a different sociocultural context, both overall and for each recommendation separately. This approach could allow for the design of more adapted and tailored strategies based on the characteristics of each patient.

In the following sections, we will discuss the results for each recommendation separately to contribute to the possibility of designing more personalized health advice.

1. Be a healthy weight

Obesity and overweight are growing health problems in most parts of the world, and also in Spain. Specifically, the prevalence of obesity in Spanish women has increased from 7.9% in 1987 to 15.5% in 2020 [32]. In our study, the prevalence of obesity was somewhat higher (18.9%), although it is important to note that weight gain is a common problem for many breast cancer survivors [33]. We observed higher compliance with this recommendation among younger women. The direct relationship between obesity and age in women, excluding individuals aged 85 and above, is well known [32]. Similarly, the inverse correlation between obesity and educational and socioeconomic levels has also been established [14,34,35]. The higher prevalence of healthy weight detected among smokers may be favored by multiple mechanisms, not all well known, and many mediated by nicotine [36]. The lower compliance with this recommendation among women with comorbidities may be influenced by limitations on the ability to exercise, thereby increasing the BMI. This is consistent with the lower compliance we also observed with the recommendation of being physically active in this group. In addition, there is evidence that certain comorbidities affecting mental health are related to a higher prevalence of obesity [37,38]. However, our study does not disaggregate the different types of morbidity, precluding us to investigating more deeply the possible effect of different pathologies.

2. Be physically active

Regular physical activity appears to be associated with reduced cancer mortality [39,40] and enhanced quality of life of survivors [41], among other beneficial effects. The percentage of sedentary women in Spain has decreased from 47% in 1993 to 32.3% in 2020 [32], but 37% of women in 2017 did not achieve the 150 min of physical activity per week recommended by the World Health Organization [42]. In our study, this figure was much higher (69%). Patients with more comorbidities were significantly less adherent. Although it seems reasonable that women with certain pathologies find it more difficult to engage in physical activity, for some conditions, it is advisable to exercise, even as part of the treatment. Adherence was also worse in women who were employed in the year before diagnosis. This result has been previously described [15], and is probably due to the reduced availability of time for self-care, as well as the lack of established workplace exercise programs in Spain (only 2.5% of individuals report exercising at their workplace) [43]. Likewise, the higher adherence detected among women without children may also be related to greater time availability. Lack of time is the main reason why 33.8% of women in Spain do not exercise [43].

3. Limit alcohol consumption

The consumption of alcohol consistently ranks as the dietary factor most strongly associated with the risk of breast cancer [44,45]. In Spain, it has been estimated that 6.2% of breast cancer cases in 2020 were attributable to alcohol consumption [46]. The number of women who habitually consumed alcohol in our country decreased until 2018. Since then, there has been a slight increase, with 3.5% of women reporting daily alcohol consumption in 2022 [47]. In our study, there was an educational gradient, with lower adherence observed in university-educated women, aligning with the alcohol consumption data in the Spanish population [32]. The lower adherence detected among working women and smokers is also in line with what has been observed at the national level [32,48], and the sociocultural factors that lead smokers to drink alcohol has been previously described [49].

4. Eat a diet rich in whole grains, vegetables, fruit and beans

The consumption of fruits and vegetables has been associated with a lower risk of developing breast cancer [44,50], although a relationship with a better prognosis in breast cancer survivors has not been demonstrated [51]. The prevalence of high compliance with this recommendation reached 51% in our participants, higher than that described in studies from other countries such as the UK [14]. In our context, the 2020 European Health Survey indicates that 71% of women reported consuming fresh fruit at least once a day, and 52% consumed vegetables, salads, or greens daily [32]. Moreover, households with a person responsible for purchasing aged over 50, especially those with retired individuals, exhibited higher fruit consumption [52]. This aligns with the higher adherence to this recommendation observed among older women, in our study and others [14]. On the other hand, reduced adherence to fruit and vegetable consumption by active smokers has also been previously reported in both Canadian [53] and Spanish adults [54].

Regarding fiber consumption, although its association with breast cancer incidence is limited [44], there is evidence suggesting that post-diagnosis dietary fiber intake decreases the all-cause mortality [26,55]. Only 4.4% of our participants complied with this recommendation, which is consistent with other studies conducted in our setting, both in long-term breast cancer survivors [15] and in the general population [56].

5. Limit consumption of “fast foods” and other processed foods high in fat, starches or sugars

The French NutriNet-Santé cohort found an 11% increase in breast cancer risk for every 10% increase in the proportion of UPFs in the diet [57]. This result was confirmed by a meta-analysis published in 2023 [58]. As for breast cancer mortality, the UK Biobank cohort showed a 16% increase for every 10% point increase in UPFs [59]. A study carried out in a representative sample of the Spanish population (ENRICA study) showed an average calorie consumption from UPFs of 24.4% [60], a figure that is very close to that found in our study (23.5%). Consistent with our results, the ENRICA study also showed a higher UPF consumption among younger women [60]. The lower consumption among mutation carriers could be related to a higher perceived risk, which would increase adherence, as has been seen in some studies on smoking [61]. However, it should be noted that our sample of mutation carriers was very small. In relation to the breast cancer molecular subtype, another Spanish multicenter study on the determinants of adherence to the WCRF/AICR recommendations in long-term breast cancer survivors also showed lower adherence to the recommendation regarding fast food consumption in women with triple-negative tumors [15].

6. Limit consumption of red and processed meat

In 2015, the International Agency for Research on Cancer classified red meat consumption as probably carcinogenic (Group 2A), linking it to the risk of colon, pancreatic and prostate cancer, and processed meat consumption as carcinogenic (Group 1), mainly based on its relationship with colorectal cancer [62]. However, some subsequent meta-analyses have also related the consumption of processed meat to the risk of breast cancer [63,64]. Meat consumption in Spain is very high, ranking seventh in the European Union in terms of per capita consumption in 2021 [65]. In our sample, only 10% of women consume less than 500 g of red meat and less than 21 g of processed meat per week, a figure very similar to other European countries [14,66] and in Spanish breast cancer survivors [15].

7. Limit consumption of sugar-sweetened drinks

Sugar-sweetened beverage consumption has been associated with an increased risk of breast cancer. A pooled meta-analysis of 27 studies revealed a 14% increased breast cancer risk in women who consumed this type of beverage [67]. In addition, a positive linear trend was observed in another study [68]. This was the recommendation with the highest adherence in our patients. More than half (54%) did not consume sugar-sweetened beverages, which is consistent with what has been observed in other studies [14,15], and in the general Spanish population, where, according to the 2014 and 2020 European Health Surveys, the percentage of women aged 55–64 years who never consumed soft drinks with sugar was 58% and 55% [32,69], respectively. The inverse association of the consumption of this type of beverage with age detected in our study has also been described in these surveys [32,69].

The use of a standardized scoring system, developed by Shams-White et al. [19] to assess adherence to the WCRF/AICR cancer prevention recommendations, improves the comparability of results across populations and countries. In addition, information on the clinical characteristics of our participants (such as stage at diagnosis, breast cancer intrinsic subtype, mutation carrier or number of comorbidities) allowed us to explore whether there were differences in adherence to recommendations according to these variables, as well as to control for these potential confounders. The estimation of prevalences and prevalence ratios standardized by several sociodemographic and clinical factors provides information about the possible independent effect of the studied factors, and contributes to the identification of population groups that can benefit from personalized intervention.

The main weakness lies in the cross-sectional nature of this study. Another limitation lies in the impossibility of comparing the adherence to the guidelines with that of women without cancer. It would have been valuable to collect information on diet and physical activity not only at diagnosis, but also at the time of patient recruitment, so that we could compare adherence to the recommendations at both times. We cannot exclude a possible survival bias since patients with a worse prognosis or worse health might have been less likely to participate. Although 80% of the participants had been diagnosed within the previous two years, it is important to recognize a possible impact of the recall bias on our results. Likewise, the data provided by the patients on their dietary habits and physical activity correspond to the year before diagnosis, although the interview was conducted after the diagnosis. This information could be influenced by the treatments received or by lifestyle modifications during the initial stages of the disease, in addition to being susceptible to social desirability bias. In addition, despite adjusting for a broad set of potential confounders, the possibility of residual confounding cannot be completely excluded. Finally, another limitation is the limited sample size when analyzing certain factors related to specific recommendations.

## 5. Conclusions

The moderate level of overall adherence to the WCRF/AICR recommendations detected highlights the need to design specific and personalized interventions to implement in women with breast cancer, paying special attention to the recommendations on total fiber, red and processed meat intake, and physical activity, as they show lower adherence. The body weight recommendation is also important to consider, because, although compliance is higher, the available evidence for its effect on prognosis is robust. Therefore, women should be counseled and supported to maintain a healthy body weight through a balanced diet and adequate physical activity. The influence of several sociodemographic and clinical variables on the degree of compliance, such as age, educational level, employment status, smoking habits, energy intake and the presence of comorbidities, emphasizes the need to address these variables when designing interventions adapted to the characteristics of these patients, with the aim of improving their quality of life and long-term prognosis.

## Figures and Tables

**Table 1:** Operationalization and adherence to the 2018 WCFR/AIRC recommendations.

				Adherence
				(N = 915)
2018 WCRF/AICR Recommendations	Operationalization		Points	n (%)
1. Be a healthy weight	BMI (kg/m^2^):			
		18.5–24.9	1	414 (45.2)
		25–29.9	0.5	300 (32.8)
		<18.5 or ≥30	0	201 (22.0)
2. Be physically active	Total moderate–vigorous physical activity (min/wk):			
		≥300	1	158 (17.3)
		150–<300	0.5	124 (13.5)
		<150	0	633 (69.2)
3. Eat a diet rich in whole grains, vegetables, fruits and beans	Fruits and vegetables (g/day):			
		≥400	0.5	465 (50.8)
		200–<400	0.25	318 (34.8)
		<200	0	132 (14.4)
	Total fiber (g/day):			
		≥30	0.5	24 (2.6)
		15–<30	0.25	483 (52.8)
		<15	0	408 (44.6)
4. Limit consumption of “fast foods” and other processed foods high in fat, starches or sugars	Percent of total kcal from ultra-processed foods:			
		Tertile 1 (<17.02)	1	305 (33.3)
		Tertile 2 (17.02–27.15)	0.5	305 (33.3)
		Tertile 3 (>27.15)	0	305 (33.3)
5. Limit consumption of red and processed meat	Total red and processed meat (g/wk):			
		Red meat <500 and processed meat <21	1	87 (9.5)
		Red meat <500 and processed meat 21–<100	0.5	329 (36.0)
		Red meat ≥500 or processed meat ≥100	0	499 (54.5)
6. Limit consumption of sugar-sweetened drinks	Total sugar-sweetened drinks (g/day):			
		0	1	498 (54.4)
		>0–≤250	0.5	389 (42.5)
		>250	0	28 (3.1)
7. Limit alcohol consumption	Total ethanol (g/day):			
		0	1	325 (35.5)
		≤14 (1 drink)	0.5	526 (57.5)
		>14 (>1 drink)	0	64 (7.0)

**Table 2:** Characteristics of breast cancer patients at diagnosis, overall and by tertiles of the WCRF/AICR score.

			Adherence to WCRF/AICR Recommendations			
		Total	Low	Moderate	High	
			(0.75–2.75)	(3.00–3.75)	(4.00–6.50)	
		n (%)	n (%)	n (%)	n (%)	*p*-Value
Total		915 (100.0)	264 (28.9)	335 (36.6)	316 (34.5)	
Adherence score, mean (SD)		3.5 (1.0)				
Age, mean (SD)		55.3 (11.4)	53.5 (11.0)	55.5 (11.4)	56.6 (11.6)	0.001
Recruiting region						
	Catalonia	477 (52.1)	150 (56.8)	179 (53.4)	148 (46.8)	0.159
	Madrid	246 (26.9)	67 (25.4)	91 (27.2)	88 (27.8)	
	Valencian Region	84 (9.2)	19 (7.2)	26 (7.8)	39 (12.3)	
	Navarre	108 (11.8)	28 (10.6)	39 (11.6)	41 (13.0)	
Educational level						
	Primary education or less	344 (37.6)	105 (39.8)	124 (37.0)	115 (36.4)	0.199
	High school/vocational training	277 (30.3)	89 (33.7)	93 (27.8)	95 (30.1)	
	University graduate	294 (32.1)	70 (26.5)	118 (35.2)	106 (33.5)	
Working status						
	No	365 (39.9)	98 (37.3)	134 (40.0)	133 (42.1)	0.498
	Yes	549 (60.1)	165 (62.7)	201 (60.0)	183 (57.9)	
Smoking status						
	No	695 (76.8)	197 (76.4)	250 (75.1)	248 (79.0)	0.491
	Yes	210 (23.2)	61 (23.6)	83 (24.9)	66 (21.0)	
Energy intake (kcal/day)						
	<1500	297 (32.5)	55 (20.8)	106 (31.6)	136 (43.0)	<0.001
	1500–1999	369 (40.3)	98 (37.1)	146 (43.6)	125 (39.6)	
	≥2000	249 (27.2)	111 (42.0)	83 (24.8)	55 (17.4)	
Menopausal status						
	Pre/perimenopausal	342 (37.4)	112 (42.4)	119 (35.5)	111 (35.1)	0.132
	Postmenopausal	573 (62.6)	152 (57.6)	216 (64.5)	205 (64.9)	
Parity						
	Parous	759 (83.0)	216 (81.8)	289 (86.3)	254 (80.4)	0.115
	Nulliparous	156 (17.0)	48 (18.2)	46 (13.7)	62 (19.6)	
Stress level						
	Low–moderate	117 (20.3)	44 (23.7)	31 (16.0)	42 (21.4)	0.158
	Substantial–high	459 (79.7)	142 (76.3)	163 (84.0)	154 (78.6)	
Number of comorbidities						
	0	443 (48.5)	130 (49.2)	159 (47.7)	154 (48.7)	0.089
	1	299 (32.7)	84 (31.8)	99 (29.7)	116 (36.7)	
	≥2	171 (18.7)	50 (18.9)	75 (22.5)	46 (14.6)	
Personal history of cancer						
	No	859 (93.9)	247 (93.6)	319 (95.2)	293 (92.7)	0.399
	Yes	56 (6.1)	17 (6.4)	16 (4.8)	23 (7.3)	
Family history of breast cancer						
	None	549 (60.0)	165 (62.5)	206 (61.5)	178 (56.3)	0.330
	Second degree only	198 (21.6)	59 (22.3)	69 (20.6)	70 (22.2)	
	First degree	168 (18.4)	40 (15.2)	60 (17.9)	68 (21.5)	
Mutation carrier						
	No	898 (98.1)	259 (98.1)	328 (97.9)	311 (98.4)	0.890
	Yes	17 (1.9)	5 (1.9)	7 (2.1)	5 (1.6)	
Years since diagnosis, mean (SD)		1.1 (1.3)	1.1 (1.3)	1.1 (1.3)	1.1 (1.3)	0.845
Tumor subtype ^a^						
	HR+	571 (68.6)	160 (67.5)	211 (69.0)	200 (69.2)	0.697
	HER2+	159 (19.1)	51 (21.5)	53 (17.3)	55 (19.0)	
	TN	102 (12.3)	26 (11.0)	42 (13.7)	34 (11.8)	
AJCC stage ^b^						
	0–I	335 (43.2)	90 (40.4)	124 (43.2)	121 (45.5)	0.698
	II	317 (40.9)	97 (43.5)	120 (41.8)	100 (37.6)	
	III–IV	124 (16.0)	36 (16.1)	43 (15.0)	45 (16.9)	

^a^ Tumor subtypes: HR+ = hormone receptor-positive tumors (estrogen receptor, ER+ and/or progesterone receptor PR+, with HER2−); HER2+ = human epidermal growth factor receptor 2-positive tumors; TN = triple-negative tumors (ER−, PR− and HER2−). ^b^ According to the 7th edition of the American Joint Committee on Cancer (AJCC) cancer staging manual [18].

**Table 3:** Standardized prevalence ratios of compliance with 2018 WCRF/AICR recommendations by participant characteristics at diagnosis.

		Standardized Prevalence Ratio (95% CI) ^a^	
		Moderate Compliance	High Compliance
Age ^b^			
	<50	1.00	1.00
	50–60	0.80 (0.61–1.05)	1.22 (0.91–1.65)
	>60	0.85 (0.60–1.20)	1.55 (1.09–2.22)
Recruiting region			
	Catalonia	1.00	1.00
	Madrid	0.96 (0.77–1.20)	1.22 (0.98–1.52)
	Valencian Region	0.82 (0.58–1.16)	1.44 (1.10–1.89)
	Navarre	0.96 (0.71–1.29)	1.18 (0.89–1.58)
Educational level			
	Primary education or less	1.00	1.00
	High school/vocational training	0.96 (0.75–1.23)	1.10 (0.87–1.39)
	University graduate	1.23 (0.97–1.56)	1.07 (0.83–1.37)
Working status			
	No	1.00	1.00
	Yes	1.04 (0.84–1.31)	1.00 (0.80–1.25)
Smoking status			
	No	1.00	1.00
	Yes	1.14 (0.93–1.39)	0.81 (0.64–1.02)
Energy intake (kcal/day)			
	<1500	1.00	1.00
	1500–1999	1.11 (0.91–1.37)	0.71 (0.59–0.86)
	≥2000	0.96 (0.76–1.23)	0.48 (0.37–0.62)
Menopausal status			
	Pre/perimenopausal	1.00	1.00
	Postmenopausal	1.23 (0.92–1.64)	0.88 (0.67–1.15)
Parity			
	Parous	1.00	1.00
	Nulliparous	0.76 (0.58–1.00)	1.15 (0.92–1.43)
Stress level			
	Low–moderate	1.00	1.00
	Substantial–high	1.26 (0.91–1.74)	0.88 (0.68–1.13)
Number of comorbidities			
	0	1.00	1.00
	1	0.99 (0.80–1.22)	1.02 (0.85–1.24)
	≥2	1.25 (0.99–1.59)	0.66 (0.49–0.89)
Personal history of cancer			
	No	1.00	1.00
	Yes	0.75 (0.48–1.18)	1.21 (0.88–1.67)
Family history of breast cancer			
	None	1.00	1.00
	Second degree only	0.94 (0.75–1.18)	1.14 (0.92–1.42)
	First degree	0.97 (0.77–1.22)	1.16 (0.93–1.44)
Mutation carrier			
	No	1.00	1.00
	Yes	0.99 (0.51–1.93)	0.95 (0.47–1.92)
Tumor subtype ^c^			
	HR+	1.00	1.00
	HER2+	0.93 (0.73–1.18)	1.00 (0.79–1.26)
	TN	1.12 (0.86–1.47)	0.88 (0.64–1.19)
AJCC stage ^d^			
	0–I	1.00	1.00
	II	1.06 (0.86–1.30)	0.95 (0.77–1.18)
	III–IV	0.97 (0.73–1.29)	1.04 (0.79–1.37)

^a^ Standardized to the overall distribution of age, recruiting region, educational level, working status, smoking status, caloric intake, parity, family history of breast cancer, number of comorbidities, menopausal status and years since diagnosis in the entire sample of breast cancer patients. ^b^ In tertiles. ^c^ Tumor subtypes: HR+ = hormone receptor-positive tumors (estrogen receptor, ER+ and/or progesterone receptor PR+, with HER2−); HER2+ = human epidermal growth factor receptor 2-positive tumors; TN = triple-negative tumors (ER−, PR− and HER2−). ^d^ According to the 7th edition of the American Joint Committee on Cancer (AJCC) cancer staging manual [18].

## Data Availability

The data presented in this study are available on request from the principal investigator. The data are not publicly available due to restrictions imposed by the ethics committees of the participating hospitals.

## References

[1] Ferlay J., Ervik M., Lam F., Laversanne M., Colombet M., Mery L., Piñeros M., Znaor A., Soerjomataram I., Bray F. (2024). Global Cancer Observatory: Cancer Today.

[2] ECIS (2025). European Cancer Information System. European Union. https://ecis.jrc.ec.europa.eu/.

[3] Allemani C., Matsuda T., Di Carlo V., Harewood R., Matz M., Nikšić M., Bonaventure A., Valkov M., Johnson C.J., Estève J. (2018). Global Surveillance of Trends in Cancer Survival: Analysis of Individual Records for 37,513,025 Patients Diagnosed with One of 18 Cancers during 2000–2014 from 322 Population-Based Registries in 71 Countries (CONCORD-3). Lancet.

[4] REDECAN Red Española de Registros de Cáncer. La Prevalencia Del Cáncer En España a 31-12-2020. https://redecan.org/es/proyectos/10/la-prevalencia-del-cancer-en-espana-a-31-12-2020.

[5] WCRF/AICR World Cancer Research Fund/American Institute for Cancer Research. Continuous Update Project Expert Report 2018. Recommendations and Public Health and Policy Implications. https://www.wcrf.org/wp-content/uploads/2021/01/Recommendations.pdf.

[6] Malcomson F.C., Wiggins C., Parra-Soto S., Ho F.K., Celis-Morales C., Sharp L., Mathers J.C. (2023). Adherence to the 2018 World Cancer Research Fund/American Institute for Cancer Research Cancer Prevention Recommendations and Cancer Risk: A Systematic Review and Meta-Analysis. Cancer.

[7] Solans M., Chan D.S.M., Mitrou P., Norat T., Romaguera D. (2020). A Systematic Review and Meta-Analysis of the 2007 WCRF/AICR Score in Relation to Cancer-Related Health Outcomes. Ann. Oncol..

[8] Inoue-Choi M., Robien K., Lazovich D. (2013). Adherence to the WCRF/AICR Guidelines for Cancer Prevention Is Associated with Lower Mortality among Older Female Cancer Survivors. Cancer Epidemiol. Biomarkers Prev..

[9] Lei Y.-Y., Ho S.C., Cheng A., Kwok C., Lee C.-K.I., Cheung K.L., Lee R., Loong H.H.F., He Y.-Q., Yeo W. (2018). Adherence to the World Cancer Research Fund/American Institute for Cancer Research Guideline Is Associated With Better Health-Related Quality of Life Among Chinese Patients With Breast Cancer. J. Natl. Compr. Canc Netw..

[10] Schroeder J., Reitz L.K., Vieira F.G.K., Da Silva E.L., Di Pietro P.F. (2023). Low to Moderate Adherence to 2018 Diet and Physical Exercise Recommendations of the World Cancer Research Fund/American Institute for Cancer Research Is Associated with Prooxidant Biochemical Profile in Women Undergoing Adjuvant Breast Cancer Treatment. Nutr. Res..

[11] Bruno E., Gargano G., Villarini A., Traina A., Johansson H., Mano M.P., Santucci De Magistris M., Simeoni M., Consolaro E., Mercandino A. (2016). Adherence to WCRF/AICR Cancer Prevention Recommendations and Metabolic Syndrome in Breast Cancer Patients. Int. J. Cancer.

[12] Tollosa D.N., Tavener M., Hure A., James E.L. (2019). Adherence to Multiple Health Behaviours in Cancer Survivors: A Systematic Review and Meta-Analysis. J. Cancer Surviv..

[13] Tollosa D.N., Tavener M., Hure A., James E.L. (2019). Compliance with Multiple Health Behaviour Recommendations: A Cross-Sectional Comparison between Female Cancer Survivors and Those with No Cancer History. Int. J. Environ. Res. Public Health.

[14] Malcomson F.C., Parra-Soto S., Lu L., Ho F., Celis-Morales C., Sharp L., Mathers J.C. (2024). Socio-Demographic Variation in Adherence to the World Cancer Research Fund (WCRF)/American Institute for Cancer Research (AICR) Cancer Prevention Recommendations within the UK Biobank Prospective Cohort Study. J. Public Health.

[15] Lope V., Guerrero-Zotano A., Ruiz-Moreno E., Bermejo B., Antolín S., Montaño Á., Baena-Cañada J.M., Ramos Vázquez M., Fernández De Larrea-Baz N., Chacón J.I. (2022). Clinical and Sociodemographic Determinants of Adherence to World Cancer Research Fund/American Institute for Cancer Research (WCRF/AICR) Recommendations in Breast Cancer Survivors—Health-EpiGEICAM Study. Cancers.

[16] Pedraza-Flechas A.M., Lope V., Vidal C., Sánchez-Contador C., Santamariña C., Pedraz-Pingarrón C., Moreo P., Ascunce N., Miranda-García J., Llobet R. (2017). Thyroid Disorders and Mammographic Density in Spanish Women: Var-DDM Study. Breast.

[17] Ruiz-Moreno E., Nuñez O., ALEVINT Group ALEVINT Software Diet Analysis Tool. https://alevint.ciberisciii.es/alevint/.

[18] Edge S.B., American Joint Committee on Cancer (2010). AJCC Cancer Staging Manual.

[19] Shams-White M.M., Brockton N.T., Mitrou P., Romaguera D., Brown S., Bender A., Kahle L.L., Reedy J. (2019). Operationalizing the 2018 World Cancer Research Fund/American Institute for Cancer Research (WCRF/AICR) Cancer Prevention Recommendations: A Standardized Scoring System. Nutrients.

[20] Shams-White M.M., Romaguera D., Mitrou P., Reedy J., Bender A., Brockton N.T. (2020). Further Guidance in Implementing the Standardized 2018 World Cancer Research Fund/American Institute for Cancer Research (WCRF/AICR) Score. Cancer Epidemiol. Biomark. Prev..

[21] Monteiro C.A., Cannon G., Levy R., Moubarac J.-C., Jaime P., Martins A.P., Canella D., Louzada M., Parra D. (2016). NOVA. The Star Shines Bright. Food Classification. Public Health. World Nutr..

[22] (2022). Ministerio de Sanidad Actividad Física Para La Salud y Reducción Del Sedentarismo. Recomendaciones Para La Población. Estrategia de Promoción de La Salud y Prevención En El SNS, Madrid. https://www.sanidad.gob.es/areas/promocionPrevencion/actividadFisica/docs/Recomendaciones_ActivFisica_para_la_Salud.pdf.

[23] Greenland S. (2004). Model-Based Estimation of Relative Risks and Other Epidemiologic Measures in Studies of Common Outcomes and in Case-Control Studies. Am. J. Epidemiol..

[24] Localio A.R., Margolis D.J., Berlin J.A. (2007). Relative Risks and Confidence Intervals Were Easily Computed Indirectly from Multivariable Logistic Regression. J. Clin. Epidemiol..

[25] Turati F., Dalmartello M., Bravi F., Serraino D., Augustin L., Giacosa A., Negri E., Levi F., La Vecchia C. (2020). Adherence to the World Cancer Research Fund/American Institute for Cancer Research Recommendations and the Risk of Breast Cancer. Nutrients.

[26] Jayedi A., Emadi A., Khan T.A., Abdolshahi A., Shab-Bidar S. (2021). Dietary Fiber and Survival in Women with Breast Cancer: A Dose-Response Meta-Analysis of Prospective Cohort Studies. Nutr. Cancer.

[27] Pierce J.P., Stefanick M.L., Flatt S.W., Natarajan L., Sternfeld B., Madlensky L., Al-Delaimy W.K., Thomson C.A., Kealey S., Hajek R. (2007). Greater Survival After Breast Cancer in Physically Active Women With High Vegetable-Fruit Intake Regardless of Obesity. JCO.

[28] Romaguera D., Ward H., Wark P.A., Vergnaud A.-C., Peeters P.H., van Gils C.H., Ferrari P., Fedirko V., Jenab M., Boutron-Ruault M.-C. (2015). Pre-Diagnostic Concordance with the WCRF/AICR Guidelines and Survival in European Colorectal Cancer Patients: A Cohort Study. BMC Med..

[29] Tollosa D.N., Holliday E., Hure A., Tavener M., James E.L. (2020). A 15-Year Follow-up Study on Long-Term Adherence to Health Behaviour Recommendations in Women Diagnosed with Breast Cancer. Breast Cancer Res. Treat..

[30] Suter F., Karavasiloglou N., Hämmig O., Rohrmann S., Pestoni G. (2023). Determinants and Changes in Adherence to the World Cancer Research Fund/American Institute for Cancer Research Cancer-Prevention Recommendations over the Past 25 Years in Switzerland. Eur. J. Cancer Prev..

[31] Demark-Wahnefried W., Aziz N.M., Rowland J.H., Pinto B.M. (2005). Riding the Crest of the Teachable Moment: Promoting Long-Term Health After the Diagnosis of Cancer. J. Clin. Oncol..

[32] INEbase Instituto Nacional de Estadística Encuesta Europea de Salud En España (EESE). Año 2020. INEbase 2025. https://www.ine.es/dyngs/INEbase/es/operacion.htm?c=Estadistica_C&cid=1254736176784&menu=resultados&idp=1254735573175.

[33] Vance V., Mourtzakis M., McCargar L., Hanning R. (2011). Weight Gain in Breast Cancer Survivors: Prevalence, Pattern and Health Consequences. Obes. Rev..

[34] Vieira L.S., Bierhals I.O., Vaz J.D.S., Meller F.D.O., Wehrmeister F.C., Assunção M.C.F. (2019). Socioeconomic Status throughout Life and Body Mass Index: A Systematic Review and Meta-Analysis. Cad. Saúde Pública.

[35] Gutiérrez-González E., García-Solano M., Pastor-Barriuso R., Fernández de Larrea-Baz N., Rollán-Gordo A., Peñalver-Argüeso B., Peña-Rey I., Pollán M., Pérez-Gómez B. (2023). Socio-Geographical Disparities of Obesity and Excess Weight in Adults in Spain: Insights from the ENE-COVID Study. Front. Public Health.

[36] Audrain-McGovern J., Benowitz N.L. (2011). Cigarette Smoking, Nicotine, and Body Weight. Clin. Pharmacol. Ther..

[37] Afzal M., Siddiqi N., Ahmad B., Afsheen N., Aslam F., Ali A., Ayesha R., Bryant M., Holt R., Khalid H. (2021). Prevalence of Overweight and Obesity in People With Severe Mental Illness: Systematic Review and Meta-Analysis. Front. Endocrinol..

[38] Chae W.R., Schienkiewitz A., Du Y., Hapke U., Otte C., Michalski N. (2022). Comorbid Depression and Obesity among Adults in Germany: Effects of Age, Sex, and Socioeconomic Status. J. Affect. Disord..

[39] Molina-Montes E., Ubago-Guisado E., Petrova D., Amiano P., Chirlaque M.-D., Agudo A., Sánchez M.-J. (2021). The Role of Diet, Alcohol, BMI, and Physical Activity in Cancer Mortality: Summary Findings of the EPIC Study. Nutrients.

[40] Tsilidis K.K., Cariolou M., Becerra-Tomás N., Balducci K., Vieira R., Abar L., Aune D., Markozannes G., Nanu N., Greenwood D.C. (2023). Postdiagnosis Body Fatness, Recreational Physical Activity, Dietary Factors and Breast Cancer Prognosis: Global Cancer Update Programme (CUP Global) Summary of Evidence Grading. Int. J. Cancer.

[41] Aune D., Markozannes G., Abar L., Balducci K., Cariolou M., Nanu N., Vieira R., Anifowoshe Y.O., Greenwood D.C., Clinton S.K. (2022). Physical Activity and Health-Related Quality of Life in Women With Breast Cancer: A Meta-Analysis. JNCI Cancer Spectr..

[42] Ministerio de Sanidad (2024). Encuesta Nacional de Salud En España 2017. Informes Monográficos: Actividad Física, Descanso y Ocio. https://www.sanidad.gob.es/estadEstudios/estadisticas/encuestaNacional/encuesta2017.htm.

[43] Ministerio de Cultura y Deporte Encuesta de Hábitos Deportivos En España 2022. https://www.csd.gob.es/sites/default/files/media/files/2022-12/Encuesta%20de%20H%C3%A1bitos%20Deportivos%20en%20Espa%C3%B1a%202022%20Resultados%20detallados.pdf.

[44] WCRF/AICR World Cancer Research/American Institute for Cancer Research. Continuous Update Project Expert Report 2018. Diet, Nutrition, Physical Activity and Breast Cancer. https://www.wcrf.org/wp-content/uploads/2021/02/Breast-cancer-report.pdf.

[45] WCRF/AICR World Cancer Research/American Institute for Cancer Research Fund. Alcoholic Drinks and the Risk of Cancer. https://www.wcrf.org/wp-content/uploads/2021/02/Alcoholic-Drinks.pdf.

[46] Rumgay H., Lam F., Ervik M., Soerjomataram I. (2021). Cancers Attributable to Alcohol.

[47] Ministerio de Sanidad Observatorio Español de las Drogas y las Adicciones. Informe 2023. Alcohol, Tabaco y Drogas Ilegales en España. Delegación del Gobierno para el Plan Nacional Sobre Drogas: Madrid, 2023. https://pnsd.sanidad.gob.es/profesionales/sistemasInformacion/informesEstadisticas/pdf/2023OEDA-INFORME.pdf.

[48] Ministerio de Sanidad Observatorio Español de las Drogas y las Adicciones. Monografía. Alcohol 2021: Consumo y Consecuencias. Delegación del Gobierno para el Plan Nacional Sobre Drogas. 2021, 109p. https://pnsd.sanidad.gob.es/profesionales/publicaciones/catalogo/catalogoPNSD/publicaciones/pdf/2021_Monografia_Alcohol_consumos_y_consecuencias.pdf.

[49] Bobo J.K., Husten C. (2000). Sociocultural Influences on Smoking and Drinking. Alcohol. Res. Health.

[50] Kazemi A., Barati-Boldaji R., Soltani S., Mohammadipoor N., Esmaeilinezhad Z., Clark C.C.T., Babajafari S., Akbarzadeh M. (2020). Intake of Various Food Groups and Risk of Breast Cancer: A Systematic Review and Dose-Response Meta-Analysis of Prospective Studies. Adv. Nutr..

[51] Peng C., Luo W.-P., Zhang C.-X. (2017). Fruit and Vegetable Intake and Breast Cancer Prognosis: A Meta-Analysis of Prospective Cohort Studies. Br. J. Nutr..

[52] Ministerio de Agricultura (2023). Pesca y Alimentación Informe del Consumo Alimentario en España. https://www.mapa.gob.es/es/alimentacion/temas/consumo-tendencias/informe_2023_baja_tcm30-685878.pdf.

[53] Palaniappan U., Starkey L.J., O’Loughlin J., Gray-Donald K. (2001). Fruit and Vegetable Consumption Is Lower and Saturated Fat Intake Is Higher among Canadians Reporting Smoking. J. Nutr..

[54] León-Muñoz L.M., Guallar-Castillón P., Graciani A., López-García E., Mesas A.E., Aguilera M.T., Banegas J.R., Rodríguez-Artalejo F. (2012). Adherence to the Mediterranean Diet Pattern Has Declined in Spanish Adults3. J. Nutr..

[55] Becerra-Tomás N., Balducci K., Abar L., Aune D., Cariolou M., Greenwood D.C., Markozannes G., Nanu N., Vieira R., Giovannucci E.L. (2023). Postdiagnosis Dietary Factors, Supplement Use and Breast Cancer Prognosis: Global Cancer Update Programme (CUP Global) Systematic Literature Review and Meta-analysis. Int. J. Cancer.

[56] González-Rodríguez L.G., Perea Sánchez J.M., Aranceta-Bartrina J., Gil Á., González-Gross M., Serra-Majem L., Varela-Moreiras G., Ortega R.M. (2017). Intake and Dietary Food Sources of Fibre in Spain: Differences with Regard to the Prevalence of Excess Body Weight and Abdominal Obesity in Adults of the ANIBES Study. Nutrients.

[57] Fiolet T., Srour B., Sellem L., Kesse-Guyot E., Allès B., Méjean C., Deschasaux M., Fassier P., Latino-Martel P., Beslay M. (2018). Consumption of Ultra-Processed Foods and Cancer Risk: Results from NutriNet-Santé Prospective Cohort. BMJ.

[58] Isaksen I.M., Dankel S.N. (2023). Ultra-Processed Food Consumption and Cancer Risk: A Systematic Review and Meta-Analysis. Clin. Nutr..

[59] Chang K., Gunter M.J., Rauber F., Levy R.B., Huybrechts I., Kliemann N., Millett C., Vamos E.P. (2023). Ultra-Processed Food Consumption, Cancer Risk and Cancer Mortality: A Large-Scale Prospective Analysis within the UK Biobank. EClinicalMedicine.

[60] Blanco-Rojo R., Sandoval-Insausti H., López-Garcia E., Graciani A., Ordovás J.M., Banegas J.R., Rodríguez-Artalejo F., Guallar-Castillón P. (2019). Consumption of Ultra-Processed Foods and Mortality: A National Prospective Cohort in Spain. Mayo Clin. Proc..

[61] Julian-Reynier C., Resseguier N., Bouhnik A.-D., Eisinger F., Lasset C., Fourme E., Noguès C. (2015). Cigarette Smoking in Women after BRCA1/2 Genetic Test Disclosure: A 5-Year Follow-up Study of the GENEPSO PS Cohort. Genet. Med..

[62] Bouvard V., Loomis D., Guyton K.Z., Grosse Y., Ghissassi F.E., Benbrahim-Tallaa L., Guha N., Mattock H., Straif K. (2015). Carcinogenicity of Consumption of Red and Processed Meat. Lancet Oncol..

[63] Farvid M.S., Stern M.C., Norat T., Sasazuki S., Vineis P., Weijenberg M.P., Wolk A., Wu K., Stewart B.W., Cho E. (2018). Consumption of Red and Processed Meat and Breast Cancer Incidence: A Systematic Review and Meta-Analysis of Prospective Studies. Int. J. Cancer.

[64] Anderson J.J., Darwis N.D.M., Mackay D.F., Celis-Morales C.A., Lyall D.M., Sattar N., Gill J.M.R., Pell J.P. (2018). Red and Processed Meat Consumption and Breast Cancer: UK Biobank Cohort Study and Meta-Analysis. Eur. J. Cancer.

[65] Food and Agriculture Organization of the United Nations (2010). Food Balances. https://www.fao.org/faostat/en/#data/FBS.

[66] Karavasiloglou N., Hüsing A., Masala G., van Gils C.H., Turzanski Fortner R., Chang-Claude J., Huybrechts I., Weiderpass E., Gunter M., Arveux P. (2019). Adherence to the World Cancer Research Fund/American Institute for Cancer Research Cancer Prevention Recommendations and Risk of in Situ Breast Cancer in the European Prospective Investigation into Cancer and Nutrition (EPIC) Cohort. BMC Med..

[67] Llaha F., Gil-Lespinard M., Unal P., de Villasante I., Castañeda J., Zamora-Ros R. (2021). Consumption of Sweet Beverages and Cancer Risk. A Systematic Review and Meta-Analysis of Observational Studies. Nutrients.

[68] Chazelas E., Srour B., Desmetz E., Kesse-Guyot E., Julia C., Deschamps V., Druesne-Pecollo N., Galan P., Hercberg S., Latino-Martel P. (2019). Sugary Drink Consumption and Risk of Cancer: Results from NutriNet-Santé Prospective Cohort. BMJ.

[69] Ministerio de Sanidad Sanidad En Datos—Encuesta Europea de Salud En España 2014. https://www.sanidad.gob.es/estadEstudios/estadisticas/EncuestaEuropea/Enc_Eur_Salud_en_Esp_2014.htm.

